# Positioning and Tracking of Multiple Humans Moving in Small Rooms Based on a One-Transmitter–Two-Receiver UWB Radar Configuration

**DOI:** 10.3390/s22145228

**Published:** 2022-07-13

**Authors:** Jana Fortes, Michal Švingál, Tamás Porteleky, Patrik Jurík, Miloš Drutarovský

**Affiliations:** 1 Department of Mathematics and Theoretical Informatics, Technical University of Košice, 042 00 Kosice, Slovakia; 2 K-Mlab Organizational Unit of Ilmsens GmbH, 040 01 Kosice, Slovakia; michal.svingal@ilmsens.com (M.Š.); tamas.porteleky@ilmsens.com (T.P.); 3 Department of Electronics and Multimedia Telecommunications, Technical University of Košice, 042 00 Kosice, Slovakia; patrik.jurik@tuke.sk (P.J.); milos.drutarovsky@tuke.sk (M.D.)

**Keywords:** UWB radar, signal processing, target positioning, target tracking, antenna height, multiple human targets, M-sequence radar

## Abstract

The paper aims to propose a sequence of steps that will allow multi-person tracking with a single UWB radar equipped with the minimal antenna array needed for trilateration. Its localization accuracy is admittedly limited, but on the other hand, thoughtfully chosen placement of antennas can increase the detectability of several humans moving in their immediate vicinity and additionally decrease the computational complexity of the signal processing methods. It is shown that the UWB radar measuring with high rate and fine range resolution in conjunction with properly tuned processing parameters can continually track people even in the case when their radar echoes are crossing or merging. Emphasis is given to the simplified method of the time-of-arrival (TOA) estimation and association and the novel method needed for antenna height compensation. The performance of the proposed human tracking framework is evaluated for the experimental scenario with three people moving closely in a small room. A quantitative analysis of the estimated target tracks confirms the benefits of suggested high antenna placement and application of new signal processing methods in the form of decreasing the mean localization error and increasing the frequency of correct target position estimations.

## 1. Introduction

UWB radar technology is still getting significant attention due to its excellent features, like high penetration ability, fine-range resolution, good environment adoption capabilities, and low power consumption. Additionally, UWB radar does not invoke personal privacy issues and there is no harmful effect on the human body as the emission power is extremely low [1]. Therefore, UWB radar shows huge potential in a wide variety of practical applications, such as see-through-wall, radar imaging, 2D/3D positioning and tracking, health monitoring, elderly care, gesture recognition, safety and security, smart home, and smart building, smart vehicles, and so on.

Numerous reviews and overviews from recent years suggest that in the case of detection and localization of people, UWB radar researchers have focused mainly on static humans concerning their vital signs [2,3,4,5,6,7]. Some meaningful references for readers interested in the field of localization and tracking of moving human targets are summarized e.g., in [7,8,9]. Seeing that the non-cooperative (tag-free) positioning of a single moving person can be considered satisfactorily mastered and well documented in the UWB radar literature, let us look at how many human targets the term multiple usually refers to. If we consider only experimentally demonstrated results and no computer simulations, it can still be a single human [10,11,12,13,14], but more probably two people in motion [15,16,17,18] or three simultaneously moving human targets [19,20,21]. This indicates that obtaining high-accuracy human tracks in crowded scenarios of a real-world complex environment is still an open research area in the UWB radar community. The indoor multipath effects, a low reflectivity of the human body, shadowing between people, overlapping of target echos in the range profiles, the wall effect in non-line-of-sight (NLOS) tracking, or the ghost target generation belong to the most challenging issues [22,23,24,25,26,27,28,29,30,31,32,33,34,35,36,37].

This paper aims to propose a sequence of steps that will allow multi-person localization and tracking with a single UWB radar equipped with the minimal antenna array needed for trilateration, i.e., one-transmitter–two-receiver configuration. Its localization accuracy is admittedly limited in comparison with spatially distributed antennas of multistatic radar [17,18,19], multiple-input multiple-output (MIMO) radar [38,39,40], or radar network [20,21,41,42,43]. On the other hand, thoughtfully chosen placement of antennas can increase the detectability of several humans moving in the small areas and additionally decrease the computational complexity of the signal processing methods applied e.g., for the deghosting solution. It will be shown that the UWB radar measuring with high rate and fine range resolution in conjunction with properly tuned processing parameters is able to continually track people even in the case when their radar echoes are crossing or merging. For that purpose first, a new version of the M-sequence UWB radar system and its antennas are described in Section 2.1. Then in Section 2.2, based on experimental measurements with the radar located at a chest height and under a room ceiling, we demonstrate the significant contribution of placing antennas at higher heights for the detection of multiple targets. In Section 2.3, the sequence of signal processing methods enabling real-time positioning and tracking of human dynamic targets will be listed. The emphasis will be given to the simplified method of the time of arrival (TOA) estimation and association (Section 2.3.1) and the novel method needed for antenna height compensation (Section 2.3.2). The performance of the proposed human tracking framework is evaluated in Section 3 for the experimental scenario with three people moving closely in a small room. Gradually adding the proposed procedures and methods, the benefits of placing antennas at a higher height, the contribution of the improved TOA estimation method and the contribution of the newly proposed method of antenna height compensation to the investigated experimental scenario are shown and quantitatively evaluated on four different signal processing combinations (Section 3.1, Section 3.2 and Section 3.3). Finally, the conclusions and future work are discussed in the last section.

## 2. Materials and Methods

The following section presents our measuring equipment and its suitable location during the measurement as well as the proposed methods for processing the measured UWB radar signals.

### 2.1. M:Explore—The Newest Version of M-Sequence UWB Radar

Maximum length binary sequence (M-sequence) and pulse radar are two commercial UWB radar systems, available e.g., in [44,45,46,47], that are widely used for human detection in the literature (e.g., [11,12,13,14,20,21,24,25,26,27,28]). Their differences are discussed e.g., in [48].

**Figure 1:**
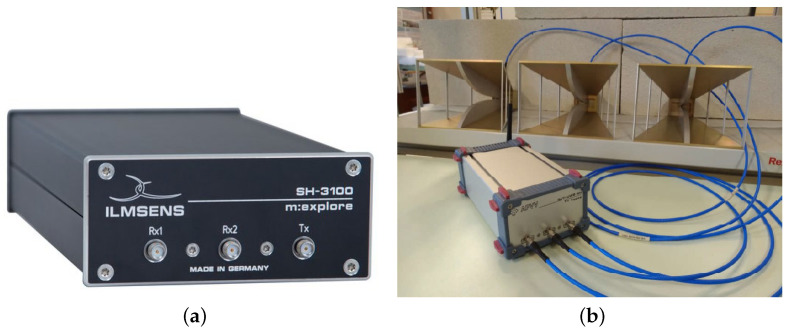
M-sequence UWB system: (**a**) an original m:explore, (**b**) a customized version of m:explore with antennas.

In our research, a custom UWB radar based on a commercial M-sequence UWB system named m:explore from the Ilmsens GmbH is used (Figure 1a) [44]. The customized version has the m:explore sensor head with embedded controller enabling data transmission in WiFi band (Figure 1b) [49]. The m:explore is an energy-efficient professional measuring device with a very high-frequency range. It was developed for a range of different measuring requirements (e.g., impedance spectroscopy, microwave imaging, time-domain reflectometry) [44]. As short-range radar, it offers few advantages in comparison with a general UWB radar such as low transmit power, harmless to humans, animals, and other machines/sensors, constantly high measurement rate, no minimum distance from the sensor to the objects (includes no reduction of the measurement rate close to the sensor), low disturbance from rain, fog or dirt due to the wavelengths used, possibility to position the sensor behind coverings as long as they are not metallic, no interference with other radars even if they are identically constructed, the possibility of synchronized multichannel systems and, therefore, reduced computing effort [44].

The minimum and maximum detection distance of a radar depend on the type of radar and antennas used, and the frequency and applicable radio regulations (which limit the transmission power). Short-range means that objects within a distance of approximately 10 m to the sensor can be detected. The higher the maximum detection range, the lower the detection accuracy in the surrounding area. In nearfield radar applications, radio regulations generally have to be met, e.g., Electronic Communications Committee (ECC) regulations. This leads to two general options for measurement electronics: 1. ECC-band, frequency range 6–8.5 GHz, conforms to ECC regulations; 2. baseband, frequency range 0.1–6 GHz, transmitted power < 1 mW. In our research, we use the baseband M-sequence short-range radar with the technical details of the transmitter and receiver summarized in Table 1 (data from [44]). Digital correlation in the embedded computer enables to suppress noise and configurable synchronous averaging helps to improve the signal-to-noise ratio. Measurement rate of the m:explore is up to 1000/s, actual maximum speed depends on the capabilities of the control computer.

The M-sequence radar has one transmitting and two receiving channels. They are connected to three double ridged waveguide horn antennas operating in the frequency band of 740 MHz to 10.5 GHz (type DRH10) [50] (Figure 1b). Their half-power beamwidth for the E plane and H plane in the dependence of used frequency can be found in [50].

### 2.2. Contribution of the High Set-Up of the Radar Antennas

According to the device provider’s recommendations [44], the radar antennas should be installed at an ideal location, which could also be behind any material, except metal, but can come into contact with the medium being examined, too. We utilize the antenna array consisting of only three elements, so they are usually placed along a horizontal line with the transmitting antenna Tx in the middle of the receiving antennas Rx1 and Rx2. As will be discussed in Section 2.3, such antenna configuration simplifies the data association as well as the computation of target coordinates by the direct method of localization. Choosing the appropriate distance between adjacent antennas is an important decision, because the bigger the distance between adjacent antennas, the better the localization accuracy [51], but on the other hand, a less clear association between the target range profiles [52].

The height of antenna placement for human detection and tracking application is usually not discussed or even not mentioned in the published experimental results, an exception is e.g., [15,26,27]. From the published measurement photos (e.g., in [10,13,20]), it can be observed that the antenna array is predominantly located at the height of the human torso, i.e., around 100–150 cm. Comfortable manipulation with the antennas at the height of the operator’s hands (or a tripod) and an extension of the radar cross-section (RCS) of a human body are the main reasons for such a choice. Our experimental results obtained for UWB radar signals measured from lower locations of the radar antennas (around 20–50 cm) have shown the same or worse tracking performance. The deterioration of the outcomes occurred probably due to the presence of furniture and other area equipment in the antenna line of sight (e.g., metal chair feet) and from more intense leg movement. On the other hand, our first experiments with the high-up placement of the radar antennas (approximately 180–210 cm) have improved the probability of target detection in the presence of two moving humans [16,26,53]. The reason is mutual shielding (shadowing) between people when a person located closer to the antennas prevents the transmission of radar signals to people further away. The shadowing effect worsens the closer someone is to the radar antennas [54].

**Figure 2:**
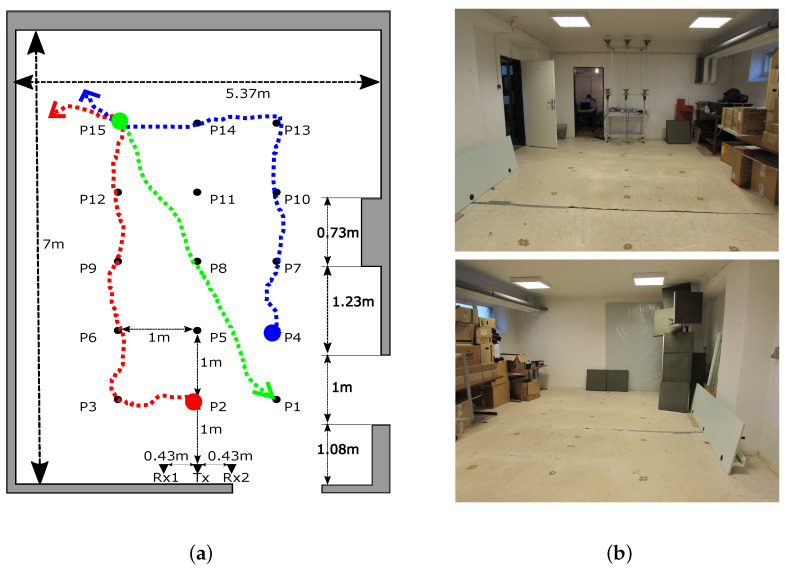
The experimental scenario: (**a**) scheme, (**b**) photos.

Since we currently own several radars and antenna arrays of the same type (Section 2.1) as well as the modular aluminum profile system enabling us to mount the antennas under the room ceiling [55], we could perform the following type of experiment shown in Figure 2. In a basement storeroom, we have built a frame on which two antenna arrays each consisting of 1 Tx and 2 Rx have been installed in two different heights: 1.3 m and 2.5 m. The horizontal distance between all Tx-Rx pairs was set to 0.47 m. The antenna arrays were connected to two m:explore radars measuring synchronously (radar system RS1 and RS2). Three people with different heights and body constitutions were moving according to predefined trajectories in the small area 2 m × 4 m placed symmetrically 1 m far from Tx. The first person (Target A) was walking from the position P2 through the position P3 to the position P15, at the same time the second person (Target B) was walking along trajectory P4-P13-P15, and after 2 s of soft movements at the position P15 the third person (Target C) was approaching from the position P15 through the position P8 to the position P1 (Figure 2).

**Figure 3:**
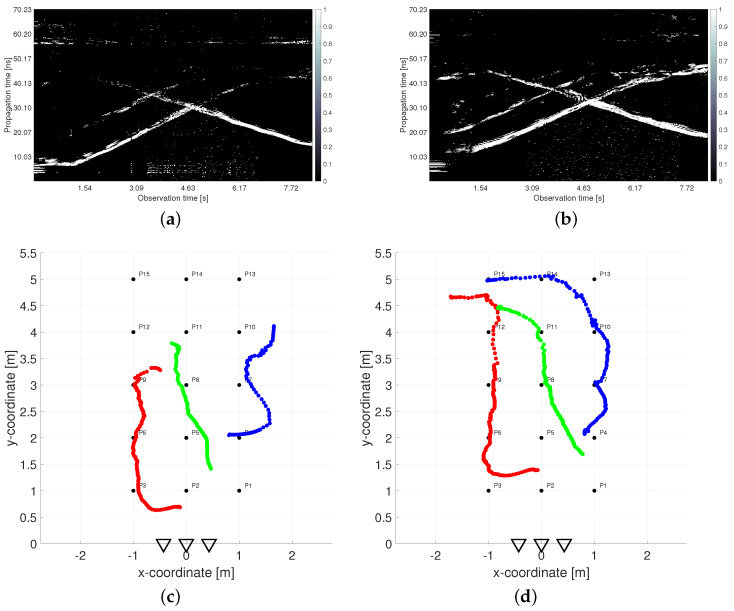
Comparison of outputs measured simultaneously in two antenna heights: (**a**) detector output from antenna height 1.3 m, (**b**) detector output from antenna height 2.5 m, (**c**) tracking output from antenna height 1.3 m, (**d**) tracking output from antenna height 2.5 m.

The negative effects of shadowing are visualized in Figure 3. The left and right columns correspond with outputs processed from UWB radar signals measured simultaneously in the height 1.3 m by RS2 and 2.5 m by RS1, respectively. The first row contains a constant false alarm rate (CFAR) detector output from the first receiving channel Rx1 of both radar systems (Figure 3a,b). The second row depicts the final tracking results obtained by the processing methods without a height compensation phase which will be further specified in Section 2.3 (Figure 3c,d). As all the used processing parameters were set to the same values for both UWB radars, the differences in the results are caused entirely by the different heights of the antennas. In the CFAR outputs, the radar echos of human targets are represented by the extended time-variant curves depicted by white color in Figure 3a,b (so-called target traces). The first and the second upward-sloping curves match with the retreating movement from antennas of Target A and Target B, respectively. At the end of the measurement, when targets were approaching the same position, their echoes were merging (Figure 3b). Target C was coming to the radar from a bigger distance, and therefore its trace is a descending curve. It crosses the traces of both remaining targets, though their tracks are separated in the measurement scenario (Figure 2). The merging and crossing target traces are very challenging tasks in radar signal processing. Despite it, the final tracks of three persons were estimated according to the real movement with acceptable localization error in the case of the high set-up of RS1 antennas (Figure 3d). The lower position of RS2 antennas resulted only in a fragmentary target track estimation (Figure 3c). It follows from the aggravated detection output (Figure 3a) in which only the target closest to the antennas was fully detected.

If we compare the detector outputs depicted in Figure 3a,b more precisely, we can remark a few more observations besides the changed detectability of target echos. In the case when the radar antennas were located under the room ceiling (Figure 3b), the rest of the crosstalk (straight lines at the bottom of Figure 3a) and the rest of the reflections from the rear wall (straight lines at the top of Figure 3a) almost completely faded. On the contrary, the time-shifted replicas of target traces partially appeared in Figure 3b (visible mostly behind Target B). However, they were suppressed during conventional signal processing and did not cause any mistake in the target count. The last and the most important difference consist in an irregular time shift of the target traces during the measurement in the higher height than the target heights are. In the lower parts of all three target, there are traces in Figure 3b, i.e., when the targets are closer to the radar. They are visibly shifted up what resulted in displaced target tracks estimated at the bottom parts of Figure 3d. The further parts of target traces in Figure 3a,b appear almost in the same propagation times (see e.g., the crossing parts). The reason for this phenomenon is the fact that the estimation of TOA and estimation of the final target coordinates is realized in two different plains. The solution to this effect is denoted as the antenna height compensation. It will be introduced for more detail in Section 2.3.

### 2.3. Signal Processing Methods Enabling Real-Time Tracking of Multiple Moving Human Targets

**Figure 4:**
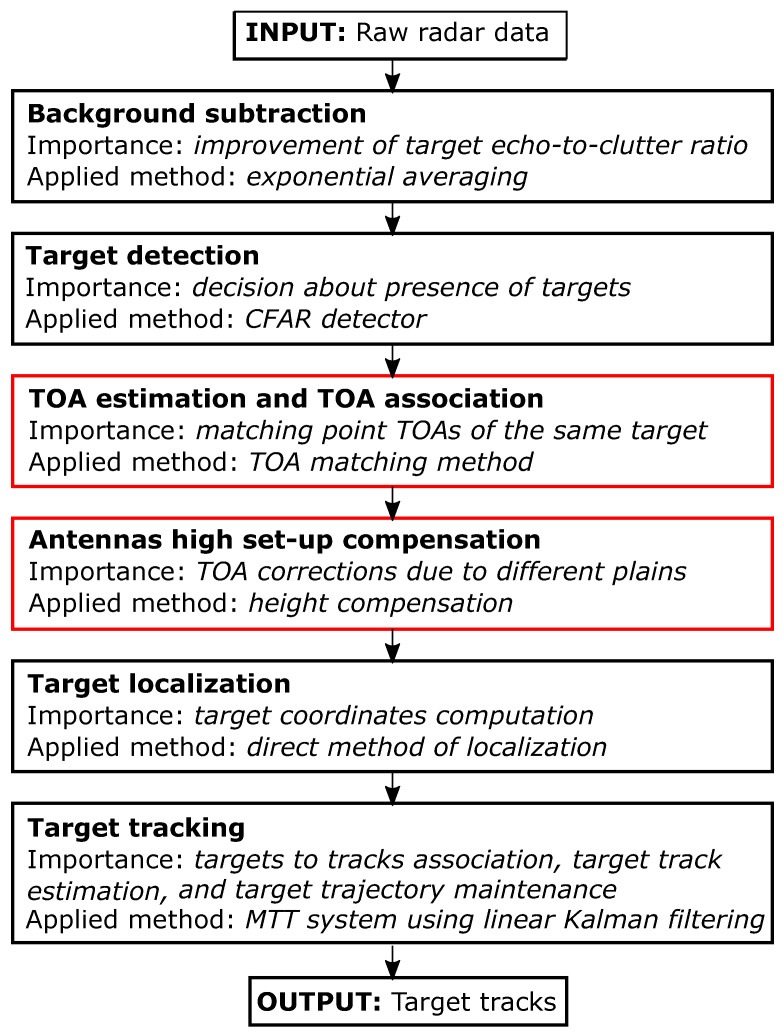
Proposed human tracking framework.

The proposed signal processing chain is introduced in the block diagram in Figure 4. In the case of considered M-sequence UWB radar, the input data have in every measurement instant form of two impulse responses of the environment, through which the electromagnetic waves emitted by the radar are propagated from Tx to Rx1 and Rx2, respectively. They are gradually processed by chosen (or newly designed) computationally effective methods of background subtraction, detection, TOA estimation, and TOA association, antennas high set-up compensation, target localization, and target tracking. The output is produced in the form of the cartesian coordinates in 2D, which create the final tracks of the dynamic human targets.

The significance of individual steps as well as applied methods in the proposed human tracking framework are listed in Figure 4 and described at length e.g., in [56]. Exceptions are the methods of TOA matching and height compensation, which represent a novelty in this processing chain. They are introduced in the following subsections. The original signal processing chain, i.e., exponential averaging, constant false alarm rate (CFAR) detector, trace connection method [52], direct method of localization, and multiple target tracking (MTT) system using linear Kalman filter, have been implemented in the application software called UWB-PerLoc-LAB enabling real-time multiple moving person tracking. It was developed in the LabVIEW environment at the Technical University of Košice, Slovakia [57]. The way of controlling communication between radar device and PC as well as the design of graphical user interface (GUI) is similar to the software UWB-StaPerLoc-LAB enabling real-time detection, localization, and tracking of static persons which is described in detail in [58]. The listed signal processing chain was even successfully modified for a real-time working wireless UWB sensor network [59]. The TOA matching and height compensation methods are currently being implemented in UWB-PerLoc-LAB software. As the first method is a simplification of the trace connection method and the second one is computationally simple, the newly designed sequence of methods can be applied to real-time tracking of moving people, too.

All the methods from the proposed human tracking framework (Figure 4) have their configuration parameters. The background subtraction parameter pExpAlpha sets the length of background estimator memory. The detection parameter pPFA determines the constant probability of false alarms. The TOA parameters (so-called allocation parameters) pSizeTarget and pMinIntegration guide the formation of the sets of points to be assigned to one or multiple targets. The height compensation parameter pTargetHeight estimates a height of the plain in which the first echoes from the moving targets are reflected. The localization parameters pLimXcor and pLimYcor define the limits of the monitored area. Finally, the MTT parameters include the state transition parameters pMinOLGI (Observation Less Gate Identification) and pMinNTI (New Target Identification) and gating parameter pG. The state transition parameters determine the speed with which each target in the allocation step starts or stops being tracked. The gating parameters establish whether the estimated coordinates can be associated with one of the existing tracks. The overall parameter settings depend on the radar measurement rate and range resolution, antennas height and their mutual distance, the size of the monitored area and its complexity, and the number of targets to be detected and tracked as well as the character of their movement. In the case of dynamic multi-person positioning and tracking, the most important parameters are the detection, TOA, and MTT parameters. The pPFA needs to be increased to detect also further targets with weak radar echoes. The pSizeTarget and pMinIntegration need to be decreased to distinguish correctly the crossing or merging traces between the targets moving at a similar distance from the Tx−Rx couple. Similarly, the pG needs to be decreased to not connect tracks of near-moving targets. It is also beneficial to increase pMinOLGI to not lose already confirmed track so easily and decrease pMinNTI to capture new targets faster. All parameters used for the measurement scenario analyzed in this article are specified in the experimental results (Section 3.1).

#### 2.3.1. TOA Matching Method

The proposed method aims to substitute every extended human target with one TOA and find such couples of TOA from Rx1 and Rx2, which correspond to the same target. In such a way, based on the assumption of a small distance between the radar antennas, it offers a simple solution to the deghosting task. The first implementation of this idea was described in [52] and successfully utilized also by different researchers, e.g. [7,13]. Now we are introducing its simplification which consists in replacing of artificial widening of potential simple targets and data association based on their connection from both receivers by direct calculation of the associated TOA couples. It means that the graphical interpretation, which was of great importance in the practical testing of this new method of association, was replaced by a quick calculation. In addition, artificial trace widening brought groups of close-spaced potential TOAs into a single target, resulting in data loss when the traces crossed or were close to each other. Now those data, particularly important for correct multi-target positioning, is preserved and if needed, complemented in a slightly different way than in the trace connection method [52]. The algorithm of the proposed TOA matching method consists of five steps from which the first three are responsible for TOA estimation (mathematically described in [52]) and the remaining two steps allow simplified TOA association:

**Step 1**  **Summation of detector output with an interval length corresponding to the assumed size of a target**
  The goal of the first two steps of the method is to eliminate the false alarms and complete the detection outputs where the target should be detected, but it has not been. The used CFAR detector produces the input data for this stage in the form of a complex binary sequence (see e.g., the columns in Figure 3a,b). The multiple reflections of electromagnetic waves from the targets and the false alarms create the set of non-zero samples. As the target size is much bigger than the radar resolution and by taking into account different shapes and properties of the human body surface, the radar echo is usually represented by clusters with a higher concentration of “1” samples. The length of such clusters is estimated by the pSizeTarget parameter. In rough approximation, it corresponds to a target size expressed in the number of samples of an impulse response according to the known radar resolution. However, if a multi-target presence is expected, a much lower value is recommended to separate their echos.
**Step 2**  **Generation of continuous TOA intervals within which the targets are detected**
  By comparison of the summation from Step 1 with pMinIntegration parameter, continuous TOA intervals for every extended target are generated. This parameter determines the minimal number of reflections in a given summation. The higher the value, the bigger the number of false alarms that are suppressed. On the other hand, the weak reflections from targets can be lost, too. As the pointed targets improperly indicated by the false alarm clusters are considerably reduced during the association phase, we recommend a smaller parameter setting.
**Step 3**  **Substitution of extended targets by simple targets**
  In this step, every continuous TOA interval is substituted by only one TOA (only one propagation time instant) representing the TOA of a potential simple target. As we suggest that the antennas be placed at a higher height during the measurement, usually, the first reflections from a moving person are from the head and shoulders and the last reflections come from the feet or are caused by multiple reflections from the floor. The TOA matching method enables to choose as point representation of the extended target, the first, middle, or the last sample of every continuous TOA interval moved in such a way to fit with the beginning of the original cluster of detected non-zero samples. As the width of the continuous TOA intervals depends on the distance of the target to the radar antennas, the positioning based on the middle or the last TOA causes a bigger localization error in the farther distances. Therefore we recommend using the first sample of continuous TOA interval as a potential simple target representation that corresponds with the leading edge of the target detection.
**Step 4**  **Creation of all potential TOA pairs that meet a matching condition**
  The association phase of the proposed approach solves the task of recognizing all couples of TOA estimated from both receivers in such a way that they produce, after the localization process, the true target positions and not the ghost targets. The main idea consists of the utilization of known and short distance *d* between adjacent antennas and results in an exactly computable and small difference between TOA estimated from both receivers (Rxi, i=1,2) and belonging to the same single target *j*. The difference is calculated based on the triangle inequality arising from the antenna layout and an arbitrary target position [52] and is labeled hereinafter as the matching condition:
  (1) $$\forall j\;|TOA_{2}^{j}-TOA_{1}^{j}|\leq \frac{2d}{c},$$
  where $TOA_{i}^{j}$ corresponds to the simple TOA estimated from the Rxi for the *j*-th target, d=dist(Tx,Rx1)=dist(Tx,Rx2), and *c* represents a light speed. The smaller the distance *d* between the antennas, the clearer the association between nearby targets. However, too small *d* worsens positioning accuracy [51].
**Step 5**  **Completion of traces based on TOA couples found in the previous time observation instant**
  This step enables completion of the potential couple of TOA by the missing TOA if only one receiving channel has detected a target echo. It is based on confirmed TOA couples found in the previous time observation instant similarly to the trace connection method. However, now the missing TOA in the actual instant is computed according to the known difference between associated TOA from the previous instant unlike simple repeating of the last known TOA.

**Figure 5:**
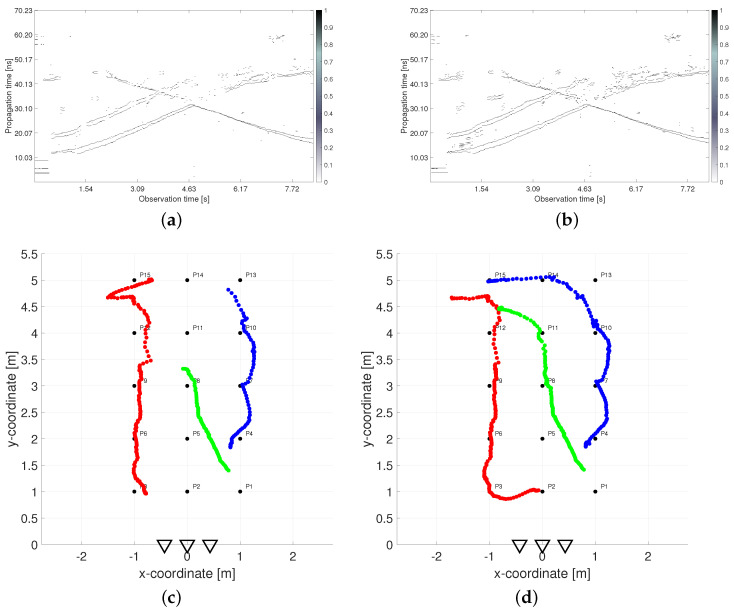
Comparison of outputs measured from antenna height 2.5 m processed by the original and the proposed TOA estimation method: (**a**) trace connection output (the original method), (**b**) TOA matching output (the proposed method), (**c**) final tracks estimated from trace connection output, (**d**) final tracks estimated from TOA matching output.

Figure 5 compares estimated traces and tracks obtained by the original trace connection method (left column) and the proposed TOA matching method (right column), respectively. As can be seen from Figure 5a,b, the TOA matching method produces richer input data for the MTT system, from which it is possible to correctly track the movement of all three people throughout the measurement period (Figure 5d). The tracks estimated after the trace connection method (Figure 5c) are also correct but incomplete in the critical parts.

#### 2.3.2. Antennas Height Compensation Method

**Figure 6:**
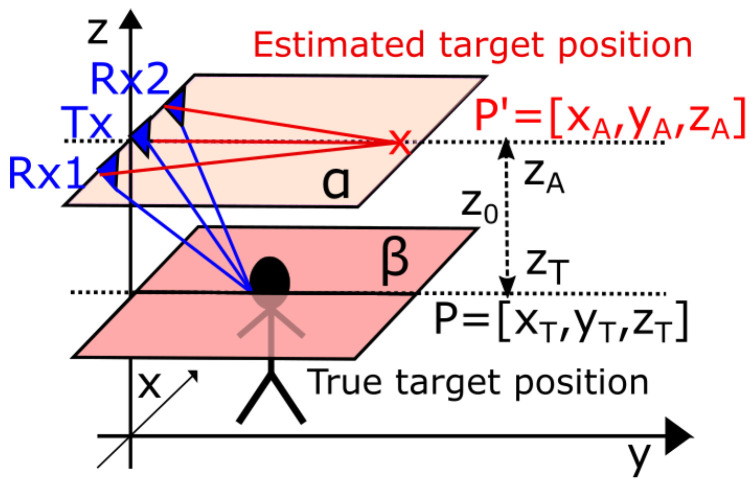
Illustration of different plains for estimation of TOA and estimation of the final target coordinates.

In Section 2.2 was shown that the high set-up of antennas increases the detectability of several humans moving in the small areas, but results in an irregular time shift of the target traces followed by an irregular shift of target tracks (Figure 3). The reason for this phenomenon is the fact that the estimation of TOA and estimation of the final target coordinates is realized in different plains (indicated by the blue and red lines in Figure 6). To solve this problem we are proposing the antenna height compensation method which decreases the TOAs obtained from the TOA matching method about the correcting value computed individually for every $TOA_{i}^{j}$, i=1,2, and all targets *j* and in such a way enable to estimate target positions more precisely.

For notation simplicity, let TOA express the measured time of electromagnetic wave propagation from the Tx towards a target *T* and reflected from the target *T* towards the Rx. From a graphical interpretation point of view, it is related to the semi-major axis *a* of the ellipse *E* with foci in Tx and Rx:

(2) $$a=\frac{c.TOA}{2}.$$

The length of the semi-minor axis *b* can be calculated by the expression

(3) $$b=\sqrt{a^{2}-e^{2}},$$

where $e=\frac{d}{2}$. To take into account the difference $z_{0}=\left|z_{A}-z_{T}\right|$ between the known antenna height $z_{A}$ and the approximated target height $z_{T}$, we need to move to 3D space. By rotating the ellipse *E* around its major axis, the spheroid *S* is obtained. Its intersection with the x−y plane at the height $z=z_{T}$ (labeled plane β in Figure 6) is a smaller ellipse $E^{\prime }$, whose semi-major axis $a^{\prime }$ relates to the searched compensated TOA value:

(4) $$a^{\prime }=\frac{c.TOA_{comp}}{2}.$$

**Figure 7:**
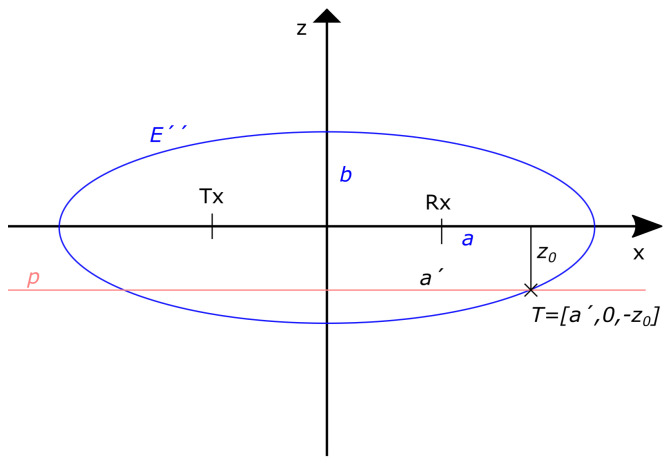
The cross-section of the spheroid *S* formed from measured TOA around couple $T_{x}-R_{x}$ and the plane β situated in the approximated target height displayed in the x−z plane.

To be able to compute $TOA_{comp}$, we consider the cross-section of the spheroid *S* and the plane β in the x−z plane for y=0 (Figure 7).

As a result, an ellipse

(5) $$E^{\prime \prime }:\frac{x^{2}}{a^{2}}+\frac{z^{2}}{b^{2}}=1,$$

and a line *p* is obtained. One of their intersection is point $T=[a^{\prime },0,-z_{0}]$, which can be substituted to the ellipse $E^{\prime \prime }$ equation:

(6) $$\frac{a^{\prime 2}}{a^{2}}+\frac{z_{0}^{2}}{b^{2}}=1.$$

From there we express

(7) $$a^{\prime }=a\sqrt{1-\frac{z_{0}^{2}}{b^{2}}}$$

and substitute known variables:

(8) $$\frac{c.TOA_{comp}}{2}=\frac{c.TOA}{2}\sqrt{1-\frac{z_{0}^{2}}{{\left(\frac{c.TOA}{2}\right)}^{2}-{\left(\frac{d}{2}\right)}^{2}}}.$$

Finally, $TOA_{comp}$ is derived based on known values $TOA,z_{0},d$, and *c*:

(9) $$TOA_{comp}=TOA\sqrt{1-\frac{4z_{0}^{2}}{c^{2}.TOA^{2}-d^{2}}}.$$

**Figure 8:**
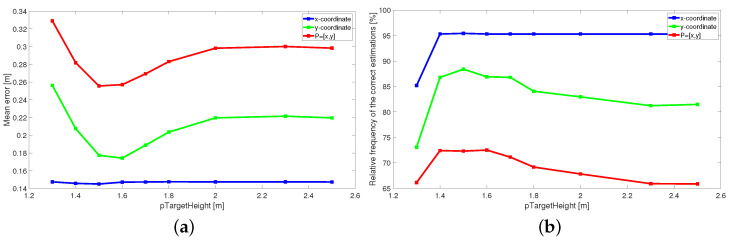
Change in the localization accuracy of *x*-coordinate, *y*-coordinate and target position P=[x,y] depending on the changing value of the input parameter pTargetHeight: (**a**) mean localization error [m], (**b**) relative frequency of the correct estimations [%].

The unknown height of targets $z_{T}$ is only roughly approximated by the method input parameter pTargetHeight. In the examined experimental scenario the real height of targets A, B, and C was 1.73 m, 1.85 m, and 1.64 m, respectively. By testing different input values of the proposed height compensation method we have found that the localization accuracy was gradually improved by decreasing the pTargetHeight parameter from the value 2 m to the value 1.6 m–1.5 m (the optimal values for the considered scenario) and then was again slightly dropping until pTargetHeight = 1.4 m (Figure 8). The application of a lower value of the parameter pTargetHeight (1.3 m and less) led to a deterioration of the localization accuracy compared to the originally measured data. Based on these findings, we recommend setting the value of pTargetHeight when tracking moving people of unknown height to approximately 1.5 m–1.6 m, which corresponds to reflections from the shoulders or upper body.

The comparison of the final tracks estimated from originally measured TOA (Figure 3d) and compensated TOA (Figure 5d) shows that the main change occurred for the *y*-coordinates up to a distance of approximately 2.5 m for all targets, but predominantly for Target A when the initial part of the trajectory was correctly shifted about 30–40 cm closer to the radar antennas. This corresponds to Figure 8, where we can see that the *x*-coordinate estimate was the most accurate estimate when calculating the relative frequency of the correct estimation as well as the mean localization error (blue lines in Figure 8). Setting the estimated target height to the recommended values reduces the mean localization error of the *y*-coordinate and at the same time increases the relative frequency of its correct estimates (green lines in Figure 8). Related to this is the same change for estimating the overall position of the target (red lines in Figure 8). The exact relations for the calculation of the plotted quantities are defined in Section 3.3.

#### 2.3.3. Trilateration Principle for a Bistatic Radar with One Tx-Two Rx Configuration

In simple terms, trilateration is a mathematical technique in which the location of a point in space is calculated using the distances from such a point to a series of known geometrical entities, e.g., a sphere or a circle. In the case of a bistatic radar, such a geometrical entity is an ellipse created around the transmitting antenna (Tx) and the receiving antenna (Rx), where Tx and Rx represent the foci of the ellipse. Since the UWB radar considered by us has one Tx and two Rx antennas located symmetrically on the same line with Tx in the middle (∥Rx1−Tx−Rx2∥=2d, *d*—usually less than 50 cm), the position of a target in 2D space can be estimated by calculating the intersections of two corresponding ellipses [60]. The simplest solution to this task is the so-called direct calculation method where we get 0, 1, or 2 estimated positions for each target [61]. In the case of two solutions, the estimated positions of the target differ only in the sign of the y-coordinate, and therefore the estimated position of the target with a positive y-coordinate is chosen, which is located in the monitored area (i.e., in front of the antennas of the radar system). In this way, we unambiguously estimate the position of the target in the monitored area based on one Tx-two Rx UWB radar configuration.

**Figure 9:**
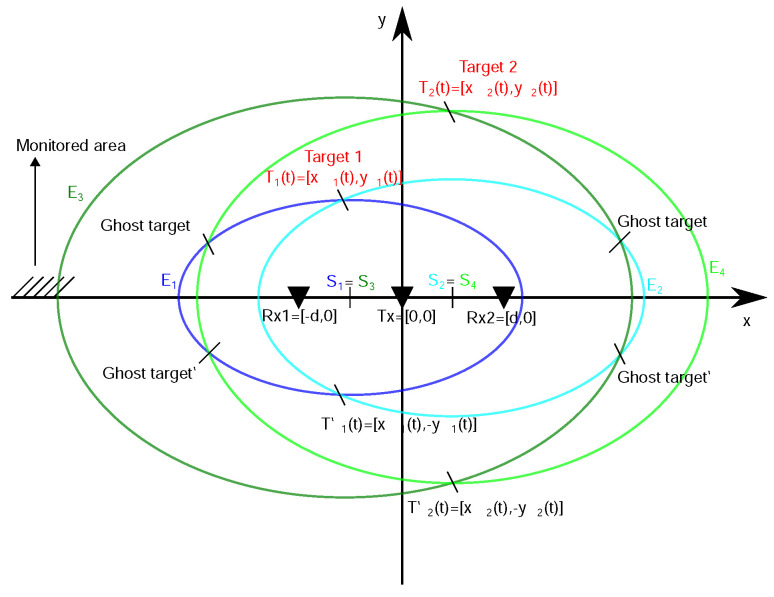
2D target localization based on one transmitter-two receiver UWB radar configuration.

The case of two-target localization by the same principle is illustrated in Figure 9. If two targets are moving in the monitored area and both receiving antennas capture their reflections, then in the TOA estimation and association phase, the proposed TOA matching method assigns one TOA to each detected distributed target and associates it with the point TOA from the second receiving antenna according to matching condition (Equation (1)). From the graphical point of view, based on estimated TOAs (Equation (2)) and (3))) from Rx1 (Rx2) the ellipses $E_{1}$ and $E_{3}$ with the same center $S_{1}=S_{3}$ ($E_{2}$ and $E_{4}$ with the same center $S_{2}=S_{4}$) are created. The matching condition is fulfilled for TOA couples corresponding to $E_{1}$ and $E_{2}$ (the ellipses of similar size) and therefore their intersection $T_{1}\left(t\right)$ represents the Target 1 location estimation in observation time instant *t*. Similarly, the matching condition is fulfilled for TOA couples corresponding to $E_{3}$ and $E_{4}$ and therefore their intersection $T_{2}\left(t\right)$ represents the Target 2 location estimation. Without correct TOA association, the intersections of all ellipse combinations are computed and in such a way also the ghost targets are obtained. In general, the ghost targets can imitate the movement of real targets for some time and therefore cannot be always reliably excluded by additional data association within the tracking phase. Their removal is then a challenging task that usually requires computationally intensive methods.

Theoretically, the trilateration principle based on described radar configuration has no limitations concerning the number of targets. However practically, the ability of passive UWB radars to detect multiple human targets is limiting. From this point of view, the proposed placement of antennas under the ceiling of the room can significantly improve the input data required for target localization.

## 3. Results

This section aims to present the performance properties of the proposed human tracking framework. To demonstrate the contribution of the described changes we analyze the experimental scenario with three people moving close to each other in a small room. The scenario scheme and photos were introduced in Section 2.2 (Figure 2).

### 3.1. Parameters

In addition to the technical parameters of the m:explore (Table 1), it is also possible to specify parameters of the whole UWB measurement system that are directly used during the processing of radar signals (Table 2).

The suggested UWB radar signal processing consists of six stages, while specific methods and their configuration parameters are listed in Table 3 (their meaning was explained in Section 2.3). These are values that have been obtained by processing a number of similar scenarios with several people moving in close proximity. If we consider applications with only one person, or with several people between which there are larger spacings, then some parameters can be set more freely, e.g., pPFA = 0.1, pSizeTarget = 15 samples, pMinOLGI = 0.33 s and pG = 3 (from 3 sigma limits). During real-time signal processing, an experienced operator can always adapt parameters according to actual conditions.

### 3.2. Qualitative Analysis

**Figure 10:**
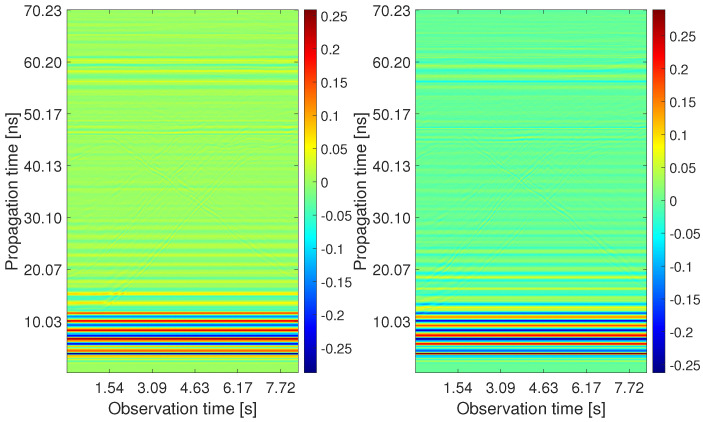
The radargrams from Rx1 and Rx2 with the raw radar data.

**Figure 11:**
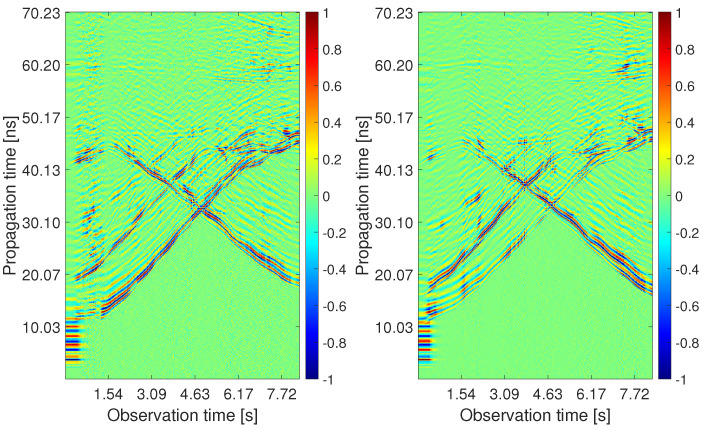
The radargrams from Rx1 and Rx2 with the subtracted background.

**Figure 12:**
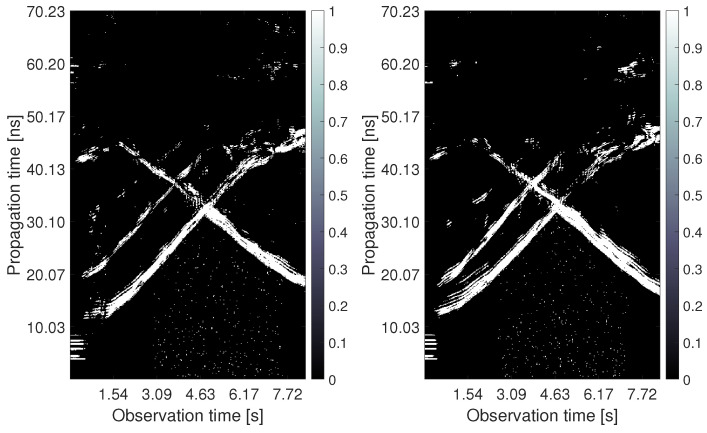
The radargrams from Rx1 and Rx2 with the detection output.

**Figure 13:**
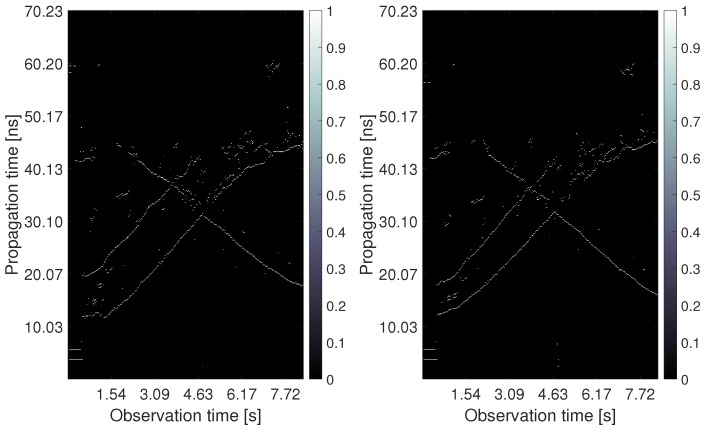
The radargrams from Rx1 and Rx2 with the estimated TOA.

**Figure 14:**
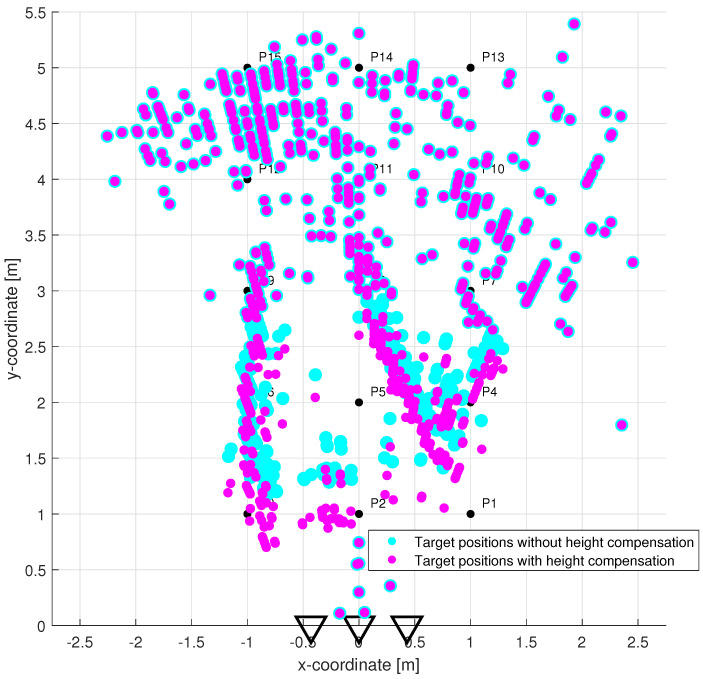
Target localization in the x−y plane without height compensation (cyan circles) vs. with height compensation (magenta circles).

**Figure 15:**
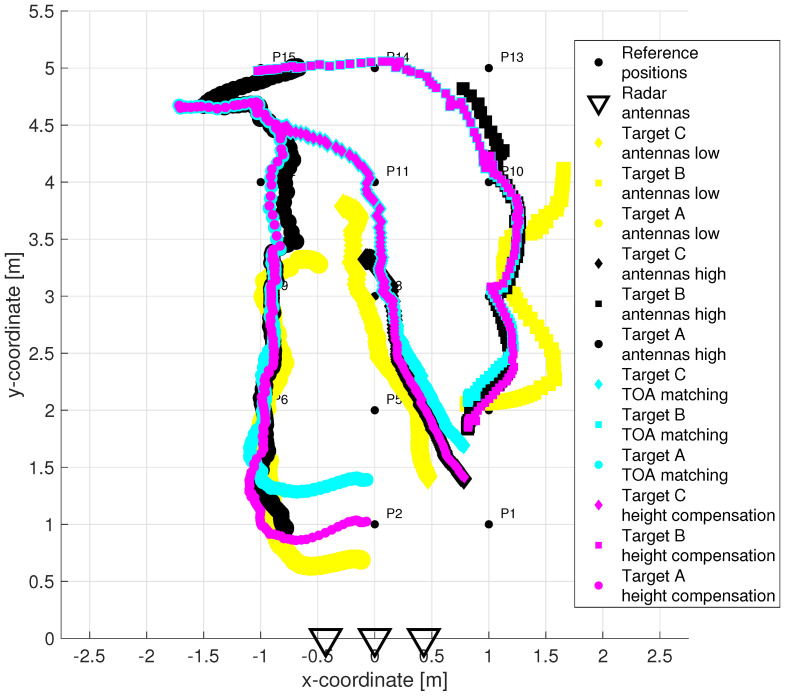
Target tracking in the x−y plane: TOA matching & no height compensation & antenna height 1.3 m (yellow tracks) vs. trace connection & height compensation & antenna height 2.5 m (black tracks) vs. TOA matching & no height compensation & antenna height 2.5 m (cyan tracks) vs. TOA matching & height compensation & antenna height 2.5 m (magenta tracks).

The processing outputs obtained for the analyzed measurement scenario are depicted in Figure 10, Figure 11, Figure 12, Figure 13, Figure 14 and Figure 15. The first four pictures are in the form of a radargram, where the *x*-axis is related to the observation time of the measurement (8.33 s in the analyzed scenario), and the *y*-axis responds to the propagation time of the impulse response. Since the radar range is much larger than the dimensions of the observed area, the original radar signals were trimmed to a propagation time of approximately 70 ns. Thanks to this, it is possible to observe the changes that occurred in the individual stages of signal processing with the naked eye.

The raw radar data from both receiving channels are depicted in Figure 10. One can observe only high-level components corresponding mainly to the direct wave between antennas. As the radar echo-to-noise and clutter ratio (ENCR) is too low, target echoes are not visible there. The level of ENCR has been increased by background subtraction (Figure 11). Now, the target echoes can be identified as two wide rising and at the end merging curves (Target A and Target B) and one decreasing curve crossing the other two (Target C). Figure 11 shows some additional high-level signal artifacts besides the target echo components. They originate mainly due to the multipath propagation of electromagnetic waves.

Since the CFAR detector had a false alarm probability set to 20%, the outputs in Figure 12 contain, in addition to target traces, some random points at the bottom of the radargram, the strongest replicas of target traces as well as direct wave remnants and a few back wall reflections. The estimated TOAs are shown in Figure 13. The proposed TOA matching method replaced each confirmed wide target echo by a point TOA and, if the matching condition was met, associated the corresponding TOA estimates from both receiving channels. Missing TOAs were also added in this step if at least one receiving channel caught a reflection from the target confirmed at the previous observation time instant (Figure 5b). In the next processing step, the associated TOAs are corrected within the height compensation method according to Equation (9). Visually, this change is reflected in a slight downward shift in the estimated TOA curves. Therefore, instead of a radargram similar to Figure 13, we show the effect of this correction on the target position estimate (Figure 14). Here the cyan and the magenta circles respond to target positions computed by the direct method of localization based on original TOA and compensated TOA, respectively. Based on the matching condition, the associated TOA couples prevented the creation of false targets (ghosts), what is confirmed by the emergence of only a few outliers in the localization stage (based on the remnants of direct wave and a short replica of Target B trace).

The results of the target tracking for four different combinations of signal processing methods are shown in Figure 15. Final tracks based on suggested high antenna placement and simultaneously applying new methods of TOA estimation and association and antenna high set-up compensation are plotted by magenta color (each target by a different symbol). The comparison of Figure 14 and Figure 15 shows that the MTT system application has allowed significantly improve the accuracy of the target localization. Tracks of three targets were correctly created and distinguished with no false and a minimum of missing positions. The initialization of the tracks took approximately 0.66 s after targets started to walk. By comparing the tracks of magenta and cyan color in Figure 15, we see the benefit achieved by the method of height compensation. It is manifested by a proper shift of trajectories closer to the antennas. By comparing the magenta and black tracks in Figure 15, a contribution of the TOA matching method versus original trace connection method can be observed. Incomplete tracks have been correctly supplemented with missing positions that correspond to crossed and merging TOA estimates (see Figure 5c,d for more details). The last comparison between the tracks of magenta and yellow color in Figure 15 makes it possible to record the contribution of the higher placement of antennas under the ceiling of the room. Poor detectability of more distant targets in the case of antenna placement in the height 1.3 m was significantly improved by increasing the position of the antennas to 2.5 m (see also Figure 3).

### 3.3. Quantitative Analysis

To provide a quantitative analysis of the results obtained for the examined scenario, we have created tolerance areas around the true positions (trajectories) of the targets in the x−y plane. The tolerance area is expressed by a circle with the center in the true position of the target and with diameter 2Δw=0.7 m, which corresponds approximately to the diameter of a human torso.

Let $T_{T}=\left(x_{T},y_{T}\right)$ and $T_{E}=\left(x_{E},y_{E}\right)$ represent the true and estimated final target position given by its coordinates in the x−y plane. Then, the estimation error of localization $e_{T}\left(\tau \right)$ at the observation time instant τ is given by

(10) $$e_{T}\left(\tau \right)=∥T_{T}\left(\tau \right)T_{E}\left(\tau \right)∥=\sqrt{{\left(x_{T}\left(\tau \right)-x_{E}\left(\tau \right)\right)}^{2}+{\left(y_{T}\left(\tau \right)-y_{E}\left(\tau \right)\right)}^{2}}.$$

The estimated positions of the targets are situated in their tolerance areas if it holds

(11) $$e_{T}\left(\tau \right)\leq \Delta w.$$

A correct estimate of the target position is confirmed, if Equation (11) holds. Based on true and estimated coordinates of persons A, B, and C as well as Equations (10) and (11), the accuracy of the target localization and tracking is evaluated using the set of quantitative characteristics stated in Table 4. There is a relative frequency of the position estimations (occurence of estimated positions of detected targets within the total number of possible target positions) and a relative frequency of the correct position estimations (according Equation (11)) distinguished.

The combinations of methods listed in Table 4 correspond to the tracks plotted in Figure 15. The first column is a summary of the quantitative characteristics calculated for the proposed measurement and signal processing procedure. The relative frequency of the estimations gained a value of almost 82%, whereas the missing values correspond in particular to the initial measurement moments needed to correctly estimate the background and confirm the initialization conditions for the new tracks. The relative frequency of the correct estimations gained a value of little more than 72%. This means that approximately 10% of the estimated positions were outside the tolerance areas. However, it is also necessary to take into account that the true trajectories of the targets were approximated at the individual observation time instants based on the known reference points through which the persons passed and on the assumption of their constant speed. If we notice that the average localization error is 25.86 cm with a standard deviation of 15.81 cm, then it would be sufficient to increase Δw to 42 cm and the relative frequency of correct estimates would increase. The maximum and minimum localization error in the case of the proposed human tracking framework was 73.83 cm and 4.33 cm, respectively. In our opinion, described values are sufficient for practical applications aimed at UWB radar tracking of moving people.

By comparing the first column with the other columns, we see the quantified benefits of each step that has been added to the framework. The worst results were obtained when measuring with an antenna height of 1.3 m—less than 50% correct estimates, mean error 47.22 cm with a standard deviation of 29.28 cm, maximum and minimum error 1.19 m and 0.12 m, respectively. The change in antenna height, the application of the new TOA matching method and the new height compensation method gradually improved the monitored characteristics (Table 4).

## 4. Discussion

In this paper, we have described in detail the procedure for measuring and processing UWB radar signals, which allowed us to significantly improve the detectability and positioning of several people moving in small rooms. We first introduced the latest version of the M-sequence radar, which measures at a speed of 32.39 impulse responses per second and with a range resolution of 2.25 cm. The radar signals measured by such a device have a form of impulse responses and therefore the proposed human tracking framework can be directly used for moving people’s positioning by employing any UWB radar measuring impulse responses with high rate and fine range resolution (e.g., impulse UWB radar).

By simultaneous experimental measurement with two radars and two sets of three antennas (Rx1-Tx-Rx2) placed at the height of the human torso and under the ceiling of the room, we demonstrated the importance of the appropriate placement of antennas on the quality of the measured signals. Expressed in numbers, the elevation of the antennas from 1.3 to 2.5 m increased the relative frequency of the target position estimates from 55% to 82% whereas the mean tracking error decreased from 47 cm to 26 cm. It was a comparative measurement, where both antenna triplets were placed on a high aluminum kit directly below each other. We assume that when we place the antennas on a bracket attached to the ceiling with a slight slope of all three antennas downwards, the beam angle of the antennas will be even better utilized and possible reflections from the ceiling will be reduced. Such an antenna placement may be advantageous e.g., for applications such as smart homes or elderly care.

In the next part of the paper, we focused on the processing of measured radar signals. We have introduced a chain of six methods that allow us to track multiple moving people in real-time. Namely, it was an exponential averaging method for background subtraction, a CFAR detector for target detection, an improved TOA estimation and association method called TOA matching, a newly proposed method of antenna height compensation, a direct localization method based on triangulation, and an MTT system using linear Kalman filtering. We discussed their configuration parameters in connection with their proper tuning for applications where more people move in the immediate vicinity. We demonstrated their functionality by correctly creating, distinguishing, and maintaining three continuous tracks corresponding to the movement of three people in the area of 2 m × 4 m. The average localization error took on the value of 26 cm with a standard deviation of 16 cm and a maximum estimation error of 74 cm. Given the size of the target, we consider these values to be sufficient for the majority of practical applications.

In the experimental results, we qualitatively and quantitatively compared the contribution of new elements in the proposed human tracking framework. The worst results were obtained when the antennas were placed at human torso height. At that time, shielding between people made it impossible to detect more distant targets, and the resulting tracks although corresponded to the movement of three people, but were considerably incomplete. An improvement in terms of the number of correct target position estimates (from 50% to 61%) as well as in terms of localization error reduction (from 47 cm to 36 cm) occurred when combining the measurement with elevated antennas (compensated by processing), but with the original TOA estimation and association method. The benefit of the improved TOA estimation and association method can be quantified if we use once the trace connection and once the TOA matching method in the same measurement and processing sequence. In such a case, the relative frequency of the correct position estimates increased from 61% to 72% whereas the mean tracking error decreased from 36 cm to 26 cm. Finally, the application of the novel antenna height compensation method has improved the relative frequency of the correct position estimates about 6% and the mean tracking error decreased about 4 cm. The true significance of even small improvements in the accuracy of target position estimates will become more apparent when such UWB radars with one transmitter-two receiver configuration are connected to the sensor network. Then it is very important that the target positions estimated from the individual nodes "fit" together, because otherwise false (multiplied) targets will be created during the tracking phase.

The presented methodology of measuring and processing UWB radar signals allows to monitor moving targets. In many applications, however, it is necessary to track people with unknown or changing physical activity, e.g., even in sleep or unconsciousness, while sitting, walking or running with stops. In such a case, it is advantageous to combine the methodology of tracking moving and static persons (based on the detection of respiratory rate). For that purpose, we presented in our last article a novel signal processing scheme for static person localization using M-sequence UWB radars [62]. A suitable criterion that allows us to switch between these two approaches is to monitor the speed of tracked people—if a person moves at a sufficient speed, we follow him/her using a methodology designed for moving targets, if his/her speed falls below a defined level, we track him/her according to respiratory movements. Such a system operating as part of a sensor network is the subject of our current research.

## Figures and Tables

**Table 1:** Technical parameters of UWB radar m:explore.

Transmitter	Receiver
UWB pseudo-noise signal	UWB analog input bandwidth: 0.1–6 GHz
No high voltage peaks, low field strength operation	RF-ports: SMA-F
Extremely stable generation driven by phase-locked RF clock (13.312 GHz)	Continuous, synchronous sub-sampling operation
Instantaneous 10 dB bandwidth 0.1–6 GHz	Extremely stable timebase derived from transmitter clock
Total output power: approx. −4 dBm	Timebase jitter < 1.5 fs (rms)
RF-port: SMA-F	Input 1 dB compression point P1dB approx. −14 dBm
Output power-down feature	System performance: > 125 dB (can be extended with external amplifiers)
Two options for ambiguity range:	Specific dynamic: 135 dB
•MLBS9:t_amb=38.4ns,r_amb=11.5m(air)	Instantaneous dynamic: >115 dB
•MLBS12:t_amb=307.6ns,r_amb=92.2m(air)	(P1 dB and 100 measurements/s)

**Table 2:** UWB radar system parameters.

Measurement System Parameter	Value
M-sequence order	12
Clock frequency	13.312 GHz
Length of impulse response	4095 samples
Range resolution	0.0225 m
Maximum range	46.11 m
Measurement rate	32.39 IR/s
Antenna spacing	0.47 m
Antenna height	2.5 m/1.3 m

**Table 3:** Signal processing parameters.

Method	Configuration Parameter	Value
Exponential averaging	pExpAlpha	0.8
CFAR detector	pPFA	0.2
TOA matching	pSizeTarget	10 samples
	pMinIntegration	3 samples
Height compensation	pTargetHeight	1.6 m
Direct localization method	pLimXcor	[−2.5 m, 2.5 m]
	pLimYcor	[0 m, 7 m]
MTT system	pMinOLGI	1 s
	pMinNTI	0.33 s
	pG	1.7

**Table 4:** Quantitative analysis of target tracks based on four different combinations of signal processing methods and measurement system set-up.

METHODS:	TOA Matching & & Height Compensation & & Ant. Height 2.5 m	TOA Matching & & without Height Compensation & & Ant. Height 2.5 m	Trace Connection & & Height Compensation & & Ant. Height 2.5 m	TOA Matching & & without Height Compensation & & Ant. Height 1.3 m
**Rel. freq. of the estimations [%]**	81.73	81.73	66.91	54.81
**Rel. freq. of the correct estimations [%]**	72.41	65.86	60.74	49.77
**Mean of $e_{T}\left(\tau \right)$ [m]**	0.2586	0.2983	0.3580	0.4722
**St. deviat. of $e_{T}\left(\tau \right)$ [m]**	0.1581	0.1623	0.2513	0.2928
**Maximum of $e_{T}\left(\tau \right)$ [m]**	0.7383	0.7496	0.9963	1.1897
**Minimum of $e_{T}\left(\tau \right)$ [m]**	0.0433	0.0655	0.0445	0.1220

## Data Availability

Not applicable.
